# A Comparative Chemogenomics Strategy to Predict Potential Drug Targets in the Metazoan Pathogen, *Schistosoma mansoni*

**DOI:** 10.1371/journal.pone.0004413

**Published:** 2009-02-09

**Authors:** Conor R. Caffrey, Andreas Rohwer, Frank Oellien, Richard J. Marhöfer, Simon Braschi, Guilherme Oliveira, James H. McKerrow, Paul M. Selzer

**Affiliations:** 1 Sandler Center for Basic Research in Parasitic Diseases, California Institute for Quantitative Biosciences, University of California San Francisco, San Francisco, California, United States of America; 2 Intervet Innovation GmbH, BioChemInformatics, Schwabenheim, Germany; 3 Laboratory of Cellular and Molecular Parasitology, Centro de Pesquisas René Rachou, Fundação Oswaldo Cruz, Belo Horizonte, Brazil; Swiss Tropical Institute, Switzerland

## Abstract

Schistosomiasis is a prevalent and chronic helmintic disease in tropical regions. Treatment and control relies on chemotherapy with just one drug, praziquantel and this reliance is of concern should clinically relevant drug resistance emerge and spread. Therefore, to identify potential target proteins for new avenues of drug discovery we have taken a comparative chemogenomics approach utilizing the putative proteome of *Schistosoma mansoni* compared to the proteomes of two model organisms, the nematode, *Caenorhabditis elegans* and the fruitfly, *Drosophila melanogaster*. Using the genome comparison software Genlight, two separate *in silico* workflows were implemented to derive a set of parasite proteins for which gene disruption of the orthologs in both the model organisms yielded deleterious phenotypes (e.g., lethal, impairment of motility), *i.e.,* are essential genes/proteins. Of the 67 and 68 sequences generated for each workflow, 63 were identical in both sets, leading to a final set of 72 parasite proteins. All but one of these were expressed in the relevant developmental stages of the parasite infecting humans. Subsequent in depth manual curation of the combined workflow output revealed 57 candidate proteins. Scrutiny of these for ‘druggable’ protein homologs in the literature identified 35 *S. mansoni* sequences, 18 of which were homologous to proteins with 3D structures including co-crystallized ligands that will allow further structure-based drug design studies. The comparative chemogenomics strategy presented generates a tractable set of *S. mansoni* proteins for experimental validation as drug targets against this insidious human pathogen.

## Introduction

Schistosomiasis is a parasitic disease infecting over 200 million people [1]. Considered a ‘neglected tropical disease (NTD)’ [2] for which, traditionally, there has been little in the way of a concerted drug discovery program, three major species of the flatworm parasite are responsible for disease in sub-Saharan Africa (*Schistosoma mansoni*, *S. haematobium*), South America (*S. mansoni*) and parts of China and South-East Asia (*S. japonicum*) [1]. Pathology associated with schistosomiasis mansoni and japonica results primarily from the accumulation of parasite eggs over the course of years and even decades giving rise initially to hepatomegaly that may be superseded by extensive liver fibrosis and possibly sequelae such as occlusion of the hepatic portal vein, portal hypertension, and gastrointestinal varices [3]. Furthermore, chronic schistosomiasis haematobia is a risk factor for squamous cell carcinoma of the bladder [4]. The disease is also known for its more subtle, and indeed underestimated [5] morbid effects, particularly in school-aged children. These include physical and cognitive under-performance, anemia and abdominal discomfort.

Despite ongoing attempts to produce a molecular vaccine [6], [7], present treatment and control of schistosomiasis relies on chemotherapy [8]. Just one drug, praziquantel (PZQ), for which the detailed mode of action is still unclear [9], [10], is widely available. Since its introduction in the late 1970's, PZQ has become the sole, WHO-recommended treatment, being safe, effective and affordable [11]. PZQ's success as a drug has contributed to a lack of urgency and investment in identifying new therapies, either in terms of chemical entities or molecular targets. This over-reliance on a single therapy to treat large populations is a serious concern regarding the potential for drug resistance [12], [13]. Resistance to PZQ has been bred on more than one occasion in the laboratory [14] and foci of transient drug resistance have been reported in the literature [15]. Thus, with the WHO-backed goal to more widely disseminate PZQ (the Schistosomiasis Control Initiative [16]), it may just be a matter of time before clinically relevant drug resistance emerges [8].

The tenuousness of therapeutic options for schistosomiasis, together with better knowledge of the molecular and biochemical idiosyncrasies of the parasite, and improved genome sequence information, are spurring increased investment in target discovery and validation [8], [17]. Further, a completely annotated *Schistosoma mansoni* genome should, in the future, provide a rich source of information for both academia and non-profit interests to identify, prioritize and prosecute drug and vaccine targets. In advance of this milestone, sufficient characterization and annotation of the genome has already taken place [18] so that in the latest Version 4 of *Schistosoma mansoni* GeneDB [19] the prediction of genes, open reading frames and translation products has been accomplished.

Given the wealth of organized data to hand, therefore, we felt it timely to put this information to work in an *in silico* comparative genomics strategy to identify a subset of schistosome genes/proteins that have potential value as drug targets in order to jump-start focused discovery efforts. Our approach was to mine the proteomes of the model organisms *Drosophila melanogaster* and *Caenorhabditis elegans* for proteins with clear sequence similarities to those in the parasite in order to identify those experimentally proven as essential, i.e., targeted gene disruption produces deleterious phenotypes (e.g. lethal, paralyzed, impaired of motility) in both model organisms. Precedence has shown that even for parasite proteins that share significant sequence similarity with vertebrate proteins, anti-parasite drugs can, nevertheless, be developed (e.g. β-tubulin, the target protein of benzimidazoles) [20]. Accordingly, the 13,283 predicted gene products of *S. mansoni* were compared in a semi-automatic process to the proteomes and phenotypic databases of *D. melanogaster* and *C. elegans* using the software Genlight [21], [22]. The output of 72 potential target proteins was manually curated leading to the identification of 35 *S. mansoni* proteins with druggable characteristics**.** Of these, 18 belong to protein families for which extensive 3D structural information is available, including bound small molecule ligands and drugs. Such structural data makes these proteins particularly suitable for prioritization of structure-based drug design strategies.

## Results

### Semi-automatic *in silico* workflows identify 72 candidate *S. mansoni* gene products

**Figure 1:**
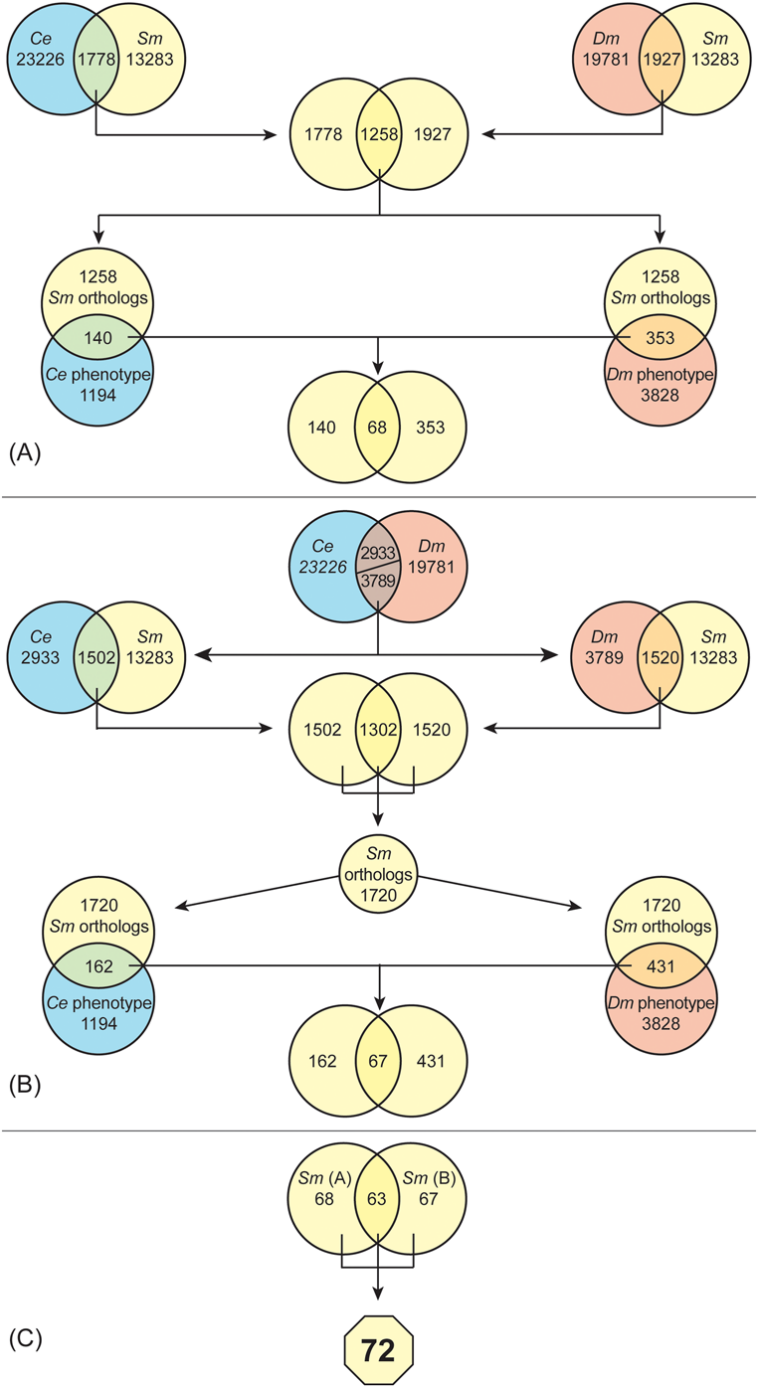
In silico workflows to identify putative drug target proteins in *S. mansoni* based on sequence and phenotype comparisons. A and B, representations of two independent workflows leading to a similar number of potential targets. C, the combination of workflows A and B generating a final number of 72 sequences (octagon) of which 63 were identical. Numbers of sequences used in each step are indicated within the respective circles. Depending on the intersection, the numbers within represent either sequence orthologs or *S. mansoni* proteins for which a deleterious phenotype is recorded in either Wormbase or Flybase. Blue, red and yellow circles display sequences from *C. elegans (Ce)*, *D. melanogaster (Dm),* and *S. mansoni (Sm)*, respectively. Details of the workflows are described in the text.

For the first *in silico* workflow, orthologs shared between the predicted proteome of *S. mansoni* [19], and the proteomes of *C. elegans* and *D. melanogaster* available at Wormbase [23] and Flybase [24], respectively, were determined (Figure 1A). For *S. mansoni* and *C. elegans,* and *S. mansoni* and *D. melanogaster*, 1778 and 1927 orthologs were identified, respectively. A subsequent comparison of both outputs demonstrated that 1258 sequences were identical. By reciprocal blastp [25], [26], this set was then compared with the phenotype databases of *C. elegans* (Caltech server) [23] and *D. melanogaster* [24], and those orthologs displaying the appropriate, pre-set, mutant phenotypes identified. The *S. mansoni* sequences of both ortholog sets were pooled and only those proteins with a 100% sequence identity were considered as potential target proteins. Altogether, 68 *S. mansoni* sequences with orthologs in *C. elegans* and *D. melanogaster* were identified.

To evaluate the performance and results of the first *in silico* approach, a second workflow was generated (Figure 1B). First, orthologs shared between *C. elegans* and *D. melanogaster* were identified. The different numbers of orthologs (2933 in *C. elegans* compared to 3789 in *D. melanogaster*) are due to the high occurrence of identical gene copies with different identifiers in Flybase. Thus, certain *C. elegans* genes generate multiple hits in *D. melanogaster*. The orthologs from both model organisms were then separately compared with the *S. mansoni* putative proteome resulting in 1502 orthologs shared between *C. elegans* and *S. mansoni,* and 1520 orthologs between *D. melanogaster* and *S. mansoni.* A redundancy check of these individual outputs established that 1302 sequences were identical. These 1302 sequences were then pooled with the extra non-redundant sequences (1502−1302 = 200 and 1520−1302 = 218 from the *C. elegans* and *D. melanogaster* comparisons, respectively) to give a total of 1720 *S. mansoni* sequences. By reciprocal blastp and using the pre-set, mutant phenotype criteria, orthologs of these sequences in the phenotype databases of *C. elegans* and *D. melanogaster* were determined. Comparison of these outputs identified 67 potential *S. mansoni* target proteins that have orthologs in both model organisms. Finally, the sequence outputs from both *in silico* workflows (68 and 67 sequences, respectively) were compared in order to determine the extent to which they were identical (Figure 1C). Sixty-three proteins were shared, thus demonstrating the reliability of the software and the compatibility of the workflows. Overall, by adding the 63 shared proteins to the 5 and 4 extra sequences exclusive to the first and second workflows, respectively, 72 *S. mansoni* proteins were considered for further manual filtering in order to delineate potential drug targets.

### Manual curation identifies 35 potential drug targets in *S. mansoni*

Manual scrutiny of the 72 sequences generated by the semi-automatic workflows was considered essential in order to remove possible redundant information and improve overall confidence in the results (Figure 1, Table 1). Each curation step is represented by a separate worksheet in Table S1.

**Table 1:** Automatic and manual filtering for potential target proteins

Filter	Number of *S. mansoni* proteins
Comparative Genomics	72
Manual Curation*	57
	
Druggable Targets based on surveys of literature and biological databases	35
3D Structure with co-crystallized ligand	18

* Removal of sequences that were redundant, not confirmed as the ortholog by reciprocal blastp, for which a phenotype was not confirmed or not expressed in the relevant life stages that persist in the human host. Numbers correspond to those on the tabs in the Microsoft Excel-worksheets provided in Table S1.

First, duplicates of four sequence entries (Smp_062300 ↔ Smp_062300.2, Smp_103470.3 ↔ Smp_103470.4, Smp_120700.1 ↔ Smp_120700.2, and Smp_138970.1 ↔ Smp_138970.4) were removed to leave 68 sequences. Subsequently, three sequences (Smp_028990.1, Smp_124240, and Smp_138970.1), not confirmed as the definitive schistosome orthologs after reciprocal blastp with both Wormbase and Flybase, were removed to produce 65 sequences. Next, seven of these sequences for which deleterious phenotypes (*e.g*. lethal, paralyzed, movement abnormal, *etc.*) could not be confirmed for the respective orthologs or for which the relevant orthologous allele or phenotype information was simply unavailable were removed. Upon scrutiny of the EST evidence (see footnote to Table S1), one additional sequence (Smp_154270) was also removed because it is only expressed in the miracidium – a developmental stage not found within the human host. The remaining 57 sequences are expressed in the relevant parasite life-stages that persist in humans, namely, the immature schistosomulum form, either prepared *in vitro* or removed from a mammalian host (*in vivo*), adult (male and/or female) and egg.

Next, each of the 57 sequences was assessed for druggability that is defined as the likelihood of being able to modulate a target's activity with a small-molecule drug [27], [28]. We mined a number of biological and literature databases to document whether orthologous proteins or proteins of the same family have been reported to be manipulated by ligands, inhibitors or even targeted by known drugs**.** For 35 of the *S. mansoni* proteins we found unambiguous evidence that homologous proteins are druggable (Table 1, Table S1). Accordingly, these might be prioritized as high value drug targets for new treatments of schistosomiasis.

Finally, we searched among the 35 *S. mansoni* proteins for homologous proteins that have a 3D structure and/or a 3D structure complexed with a ligand, inhibitor or drug. Such structural information would enhance the druggability value by facilitating a structure-based drug design strategy, including homology modeling, docking, virtual screening or pharmacophore-based screening [29], [30]. Eighteen of the 35 *S. mansoni* proteins fulfilled these conditions (Tables 1 and 2) and another 8 had at least partial 3D structure information available (Table S1).

**Table 2:** Druggable target proteins belonging to protein families with 3D structural information, including co-crystallized ligands

*S. mansoni* Putative Protein	Molecular Function GO Annotation at *Schistosoma mansoni* GeneDB [19]	GeneDB Accession Number
GTP-binding protein	GTP binding, signal transducer activity	Smp_005790
Glycogen synthase kinase 3 related	ATP binding, protein kinase activity	Smp_008260.1
Methionine amino peptidase	Metallo exopeptidase activity	Smp_011120
Calmodulin dependent protein kinase II	Protein kinase activity	Smp_011660.2
Protein phosphatase-2a	Hydrolase activity	Smp_030710
Nuclear transport factor	Transporter activity	Smp_037700
Vesicular-fusion protein nsf	ATP-binding, nucleoside triphosphatase activity, nucleotide binding	Smp_057320
Rac GTPase	GTP-binding	Smp_062300
Elongation factor tu	GTP-binding, translation elongation factor activity	Smp_073500.1
Neuroendocrine convertase	Subtilase activity	Smp_077980
Myosin heavy chain	Motor activity	Smp_085540.6
Nucleoside diphosphate kinase	ATP-binding, nucleoside diphosphate kinase activity	Smp_092750
Rab GDB-dissociation inhibitor	Rab GDP-dissociation inhibitor activity	Smp_094420
Heat shock protein 70	ATP-binding	Smp_106130.2
N-myristoyl transferase	Glycylpeptide N-tetradecanoyltransferase activity	Smp_121420
Choline o-acyltransferase	Acyltransferase activity	Smp_146910
Rab 6	GTP-binding	Smp_163580
Amino acid decarboxylase	Carboxy lyase activity	Smp_171580

Additional information such as key references reviewing druggability are available in Supplementary Table S1.

## Discussion

The comparative chemogenomics strategy described herein provides a prioritized and testable list of potential target proteins for *S. mansoni*, a metazoan pathogen causing chronic and debilitating disease in humans. The intent was first to mine the *S. mansoni* genome and identify putative essential genes based on similarity to experimentally-determined essential genes/proteins in two model metazoans and then define a subset of potential drug targets for which structural information of known target proteins, including bound ligands, exist. Both the strategy and outputs are in keeping with the recent establishment of a TDR Drug Targets Prioritization Database [31] by the World Health Organization's Special Programme for Research and Training in Tropical Diseases that facilitates the identification and prioritization of target genes in a number of pathogenic organisms, including those responsible for NTDs.

A number of factors were considered during the development and execution of the workflows in order to provide both confidence in the data generated and a solid platform from which to predict the druggability of individual *S. mansoni* proteins. First, rather than comparison with one metazoan proteome we incorporated two into the analysis, particularly as both *C. elegans* and *D. melanogaster* are phylogenetically remote from *Schistosoma*. Secondly, we only considered those orthologous genes/proteins for which phenotypes were generated via targeted gene mutagenesis. We discounted orthologs with phenotypes arising from RNAi (RNA interference) due to the potential for non-specific, off-target effects and false positive results including with *Drosophila* cells [32], [33], [34]. Nevertheless, the present protocol can be adapted to include RNAi phenotypes should one wish to cast the net wider. As a final stricture in the analysis, we selected for severely deleterious phenotypes such as death or those involving motility disorders with the aim of producing both a short list of testable targets and enriching for phenotypes that should be obvious when targeting the respective schistosome genes by chemical and/or genetic means (see below). We would state that our strategy focuses on potential targets based on loss-of-function and that gain-of-function targets such as those involved in drug agonism e.g., ion channels, will be missed. As many current anthelmintics, including PZQ, are agonists, it would be worthwhile developing systems for gain-of-function mutants in model organisms (for example using targeted overexpression banks).

The present strategy differs from previous comparative genomic screens for infectious diseases and for which the goal was to identify potential drug targets unique to the pathogen based on a user-prescribed similarity cut-off value in order to decrease the potential for toxicity of any new chemotherapeutic [35], [36]. Though this ‘exclusion’ approach is sound, nevertheless, examples abound of pathogen proteins that possess considerable similarity to human proteins and yet are valid drug targets e.g., parasite cysteine proteases [37], [38], [39], and the remarkable example of the highly conserved β-tubulin protein, the target of benzimidazoles [20], even though they might have failed a user-prescribed similarity cut-off as part of an exclusion genomics screen [35]. In addition, it is often the case that drug selectivity for a pathogen arises due to idiosyncrasies in pathogen physiology such as slower turnover of the target protein allowing for more pronounced drug action, e.g., ornithine decarboxylase in *Trypanosoma brucei,* the causative agent of African Sleeping Sickness [40], differences in protein regulation, e.g., dihydrofolate reductase in *Plasmodium falciparum* [41] or a lack of functional redundancy compared to the host [42]. Thus, we consider that the present similarity-based strategy to mine the *S. mansoni* genome represents a useful contribution to the consideration of new anti-schistosomal therapeutic targets.

An important aspect of developing drugs to NTDs such as schistosomiasis and for which profits are nil or marginal at best, is the need to keep costs of drug development to a minimum. Experience has shown that drug development programs for pathogen-specific targets of often unknown function, although a valid scientifically, are, in many cases, more expensive. Our strategy, therefore, to identify homologous rather than pathogen-specific potential targets is intended to reduce costs by leveraging the biochemical, chemical and structural data and tools already available for therapeutic targets of proven value in other clinical contexts, *i.e.,* ‘piggy-back’ drug discovery [43], [44], [45]. The finding that 35 of the *in silico*-identified 72 sequences are indeed homologous to known druggable targets supports our strategy. Moreover, 18 of these belong to protein families for which 3D structural information including ligands or even drugs is available (e.g., N-(4-Methoxybenzyl)-N′-(5-Nitro-1,3-Thiazol-2-YI)Urea and Carbidopa). This opens the route not only for classical biochemical studies but also for structure-based drug discovery approaches [29], [46], [47]. For instance, among the 18 candidate proteins, small molecule scaffolds exist for methionine aminopeptidase targeting cancer [48] and malaria [49], N-myristoyl transferase against fungal infections [50] and Rac-GTPase against cancer [51]. The availability of specific small molecules adds incentive to prioritizing the experimental validation of the orthologous schistosome proteins.

Unlike for both model organisms, standardized functional genomic tools, such as targeted gene disruption or gene ‘knock-in’ technology, are not yet established for schistosomes. This represents a stumbling block for interrogating gene function with confidence. However, transient RNAi with double stranded RNAi or small interfering (si)RNA has gained a foothold as a useful technique for gene knock down (if not knock out), including in those developmental stages relevant to infection and pathology in humans (somules, adults and eggs) [52], [53]. We would assume, therefore, that transient RNAi will be an important tool to experimentally establish gene essentiality until more rigorous reverse genetic techniques become established. Where available, a complementary strategy to RNAi would be to employ a chemical genetics approach using protein-selective small molecules (see examples above). Such an approach has already been validated regarding schistosome cysteine proteases [37] and enzymes involved in redox metabolism [54].

We have mined and compared the predicted proteome of the metazoan pathogen, *S. mansoni*,with those of two well-studied model organisms in order to identify potential drug targets. The chemogenomics strategy has produced a tractable list of prioritized genes for further investigation, one or more of which might contribute to badly-needed drug discovery programs for this prevalent human disease.

## Methods

### Datasets

All protein datasets are available on Flybase (version FB2006_01) [24], Wormbase release 195 [23], and *Schistosoma mansoni* GeneDB v4.0 [19]. The *D. melanogaster* phenotype database was generated using the Flybase QueryBuilder. All proteins with the mutant phenotypes “lethal” and/or “neurophysiology defective” were downloaded. Similarly, the *C. elegans* phenotype database was generated using the Caltech server based on Wormbase release 177 [23]. All proteins with the mutant phenotypes “lethal”, “paralyzed”, “movement abnormal” and/or “muscle system physiology abnormal” were downloaded. However, only data from knock-out mutants (alleles) were used; RNAi phenotypes were excluded due to the potential for non-specific effects [32], [33], [34].

### Comparative Genomics using Genlight

All genome comparisons performed were based on translated genomes using the software Genlight [21], for which a public WWW-server is available at the University of Bielefeld, Germany [22]. Genlight is a client/server based program suite developed for large scale sequence analyses and comparative genomics calculations. A key functionality of Genlight is the determination of orthologs via reversed or reciprocal blast searches. Sequences from organism A are compared with those from organism B and *vice versa* using the respective blast program [25], [26]. Orthologous sequences are then defined as those best hit sequences that find each other in such bidirectional blast searches. The sequence alignment overlap and the E-value cut off can be preset in accordance with the goal of the experiment [21], [29]. Within Genlight, we defined orthologs as best reciprocal blastp hits with a minimum of 70% sequence alignment overlap and an E-value of 0.01 or smaller.

A useful feature of the Genlight software is its user friendliness in the post-processing of results including the employment of predefined filters and operations. For example, result sets can be re-used directly to exclude duplicates in order to generate non-redundant datasets. This feature has been employed as described in the two *in silico* workflows (Figure 1) and resulted in the identification of 72 potential target proteins in the *S. mansoni* predicted proteome (Table S1) for which deleterious phenotypes were identified for the respective orthologs in both *C. elegans* and *D. melanogaster*.

### Manual curation and filtering for potential target proteins

After executing two separate electronic workflows (see Figure 1), we manually curated the schistosome protein sequence output for consistency with our goal of identifying potential drug targets. First, redundant sequences were removed from the dataset. Further curation included removal of sequences (i) not confirmed as orthologs by reciprocal blastp searches with both Wormbase and Flybase, (ii) for which targeted gene disruption of the appropriate ortholog does not yield a deleterious phenotype, and (iii) which, based on the available EST evidence, are not expressed in the relevant parasite developmental stages infecting humans (i.e., schistosomulum (immature worm) either prepared *in vitro* or removed from a mammalian host (*in vivo*), adult (male and/or female) and egg). Consideration was also given to the potential druggability of the parasite protein by manually mining biological and literature databases on the internet including DrugBank [55], [56], UniProt [57], [58], Prosite [59], [60], OMIM [61], [62], InterPro [63], [64] and Pfam [65], [66]. Druggability was defined as the likelihood of being able to modulate the activity of the protein target with a small-molecule drug [27], [28]. Finally, the PDB database [67] was searched for 3D structural information of proteins homologous to each parasite protein, including co-crystallized ligands. The 72 sequences, together with the subsequent manual curation steps, are presented in Table S1.

## Supporting Information

Table S1 This table shows the output and manual curation of potential S. mansoni drug targets generated by comparative genomics with Caenorhabditis elegans and Drosophila melanogaster. The table comprises 7 worksheets each with a different level of information for the respective sequence lists. Leftmost in the table, the first worksheet contains the 72 sequences generated from the two separate electronic workflows utilizing the genome comparison software, Genlight. Each worksheet thereafter represents a subsequent manual curation step involving the removal of sequences (i) that are redundant (remaining 68 non-redundant entries), (ii) that are not confirmed as orthologs by reciprocal blastp searches with both Wormbase and Flybase (remaining 65 confirmed orthologs), (iii) for which targeted gene disruption of the appropriate ortholog does not yield a deleterious phenotype as declared in both Wormbase and Flybase (remaining 58 confirmed phenotypes), (iv) that are not expressed in the relevant developmental stages of the parasite infecting humans (remaining 57 expressed in the relevant life stage; for an explanation of how this was performed see below), (v) that are not druggable as indicated by manual mining of the biological and literature databases available on the internet (remaining 35 druggable targets), and (vi) for which homologous proteins with 3D structural information (including co-crystallized ligands) was not found (remaining 18 proteins with both a 3D structure and co-crystallized ligand). To determine in which developmental stage a putative protein is expressed, the following procedure was performed. In the S. mansoni genome browser [68], the sequence name (e.g., Smp_000040) is imputed into the text field, “landmark or region”. On the returned search page, the graphic produced by the latest GeneDB working model and displaying the organization of the gene is clicked to reveal a variety of information, including exon/intron junctions, physicochemical characteristics of the putative protein and gene ontology. Activating the hyperlink “DNA” reveals the unspliced DNA, spliced DNA and amino acid sequences. Next, a blastn search at NCBI of the spliced sequence is performed via the hyperlink “Send to BLAST at NCBI”. The analysis is constrained using the search set ‘non-human, non-mouse ESTs (EST_others)’, the organism ID number of 6183 for S. mansoni (taxid:6183) and the program selection set to ‘somewhat similar sequences’. On the EST list returned, each accession is scrutinized for its life-stage origin by activating the relevant link. The lowest maximum score accepted for consideration was 250. Lower scores tended to be too short to be reliably ascribed to the gene under study.
(0.26 MB XLS)
